# An acidic, thermostable exochitinase with β-*N*-acetylglucosaminidase activity from *Paenibacillus barengoltzii* converting chitin to *N*-acetyl glucosamine

**DOI:** 10.1186/s13068-014-0174-y

**Published:** 2014-12-10

**Authors:** Xing Fu, Qiaojuan Yan, Shaoqing Yang, Xinbin Yang, Yu Guo, Zhengqiang Jiang

**Affiliations:** Department of Biotechnology, College of Food Science and Nutritional Engineering, China Agricultural University, Beijing, 100083 China; Bioresource Utilization Laboratory, College of Engineering, China Agricultural University, Beijing, 100083 China

**Keywords:** Chitinase, Exochitinase, *Paenibacillus barengoltzii*, Chitin conversion, *N*-acetyl-β-glucosamine

## Abstract

**Background:**

*N*-acetyl-β-D-glucosamine (GlcNAc) is widely used as a valuable pharmacological agent and a functional food additive. The traditional chemical process for GlcNAc production has some problems such as high production cost, low yield, and acidic pollution. Hence, to identify a novel chitinase that is suitable for bioconversion of chitin to GlcNAc is of great value.

**Results:**

A novel chitinase gene (*PbChi74*) from *Paenibacillus barengoltzii* was cloned and heterologously expressed in *Escherichia coli* as an intracellular soluble protein. The gene has an open reading frame (ORF) of 2,163 bp encoding 720 amino acids. The recombinant chitinase (PbChi74) was purified to apparent homogeneity with a purification fold of 2.2 and a recovery yield of 57.9%. The molecular mass of the purified enzyme was estimated to be 74.6 kDa and 74.3 kDa by SDS-PAGE and gel filtration, respectively. PbChi74 displayed an acidic pH optimum of 4.5 and a temperature optimum of 65°C. The enzyme showed high activity toward colloidal chitin, glycol chitin, *N*-acetyl chitooligosaccharides, and *p*-nitrophenyl *N*-acetyl β-glucosaminide. PbChi74 hydrolyzed colloidal chitin to yield *N-*acetyl chitobiose [(GlcNAc)_2_] at the initial stage, which was further converted to its monomer *N*-acetyl glucosamine (GlcNAc), suggesting that it is an exochitinase with β-*N*-acetylglucosaminidase activity. The purified PbChi74 coupled with RmNAG (β-*N*-acetylglucosaminidase from *Rhizomucor miehei*) was used to convert colloidal chitin to GlcNAc, and GlcNAc was the sole end product at a concentration of 27.8 mg mL^-1^ with a conversion yield of 92.6%. These results suggest that PbChi74 may have great potential in chitin conversion.

**Conclusions:**

The excellent thermostability and hydrolytic properties may give the exochitinase great potential in GlcNAc production from chitin. This is the first report on an exochitinase with *N*-acetyl-β-D-glucosaminidase activity from *Paenibacillus* species.

## Background

Chitin is a β-1,4-linked linear insoluble polymer of *N*-acetyl D-glucosamine (GlcNAc), which is an abundant polysaccharide, after cellulose. Chitinolytic enzymes have been classified into two categories according to their methods of cleavage on chitin chains. Endochitinases (EC 3.2.1.14) randomly catalyze the cleavage of β-1,4-glycosidic bonds in chitin to release *N*-acetyl chitooligosaccharides (*N*-acetyl COSs); exochitinases (EC 3.2.1.52) can be divided into two subcategories: chitobiosidases (EC 3.2.1.29), which catalyze the successive release of diacetylchitobiose units in a stepwise fashion as the sole product from chitin, and *N*-acetyl β-1,4-D-glucosaminidases (EC 3.2.1.30), which act as an exo-splitting model on diacetyl chitobioses and *N*-acetyl COSs (the formerly classified EC 3.2.1.29 and EC 3.2.1.30 have been included in EC 3.2.1.52) [1]. They have received much attention in recent years due to various applications such as production of functional *N*-acetyl COSs and GlcNAc [2], bioconversion of chitin waste to bioethanol [3], and bio-control of fungal phytopathogens [4].

Chitinases are mainly classified into the glycoside hydrolase (GH) families 18 and 19, and widely exist in a great range of organisms including bacteria, fungi, plants, insects, and animals [5]. Among them, bacterial chitinases gained more research interest due to their diverse properties and potential industrial applications [6]. To date, a number of bacterial chitinases have been isolated and characterized [3,7-15]. Many chitinase genes have also been cloned and expressed from bacteria such as *Bacillus* sp. DAU101 [16], *B. licheniformis* [17], *Paenibacillus* sp. [18], *Sanguibacter antarcticus* [19], and *Stenotrophomonas maltophilia* [4]. However, most reported chitinases belong to endochitinases, while a few exochitinases were found in bacteria such as *Thermococcus chitonophagus* [7], *Microbispora* sp. [20], and *Bacillus* sp. [16]. Until now, *Paenibacillus* species have been reported to produce some endochitinases [3,11-14,18,21], but no exochitinase from *Paenibacillus* species has been found.

GlcNAc has attracted much attention in recent years due to its therapeutic activity in osteoarthritis. In particular, the demand for GlcNAc in the health food industry is growing rapidly [22]. Generally, GlcNAc was commercially prepared via an extreme process based on the high concentration acid hydrolysis of chitin at relatively high temperatures. But the process has some problems, such as high production cost, low yield, and acidic pollution [22]. Besides, GlcNAc production by chemical methods limits GlcNAc’s application in the food industry [23]. Hence, several attempts have been performed on the GlcNAc production using enzymatic hydrolysis of chitin [24,25], but the yield still remains low. Thus, further development of enzymatic chitinolysis for GlcNAc production is required [11].

Recently, a novel species of *Paenibacillus*, *viz.*, *Paenibacillus barengoltzii*, has been reported [26]. We have newly isolated a thermophilic marine bacterium *Paenibacillus barengoltzii* CAU904 from the South China Sea which can grow at 50°C. Here, we report gene cloning, expression, and biochemical characterization of a novel exochitinase (PbChi74) from this strain. The conversion of chitin to GlcNAc by the enzyme coupled with the β-*N*-acetylglucosaminidase (RmNAG) from *Rhizomucor miehei* [27] was further evaluated. This represents the first report on an exochitinase with β*-N*-acetylglucosaminidase activity from *Paenibacillus* species.

## Results

### Cloning and sequence analysis of a chitinase gene from *P. barengoltzii*

A 375-bp gene fragment was obtained by PCR using degenerate primers ChiF and ChiR with *P. barengoltzii* CAU904 genomic DNA as the template. This sequence was subsequently used to design hiTAIL-PCR primers (Table 1) to amplify the full-length coding region of the gene. The full-length chitinase gene (*PbChi74*) has an open reading frame (ORF) of 2,163 bp encoding 720 amino acids with a theoretical molecular mass of 74.2 kDa and *p*I of 4.74. A signal peptide of 34 amino acids was predicted by SignalP analysis.

**Table 1:** **Conditions and primers for hiTAIL-PCR**

**Reaction**	**Numbers of cycles**	**Thermal settings**
**Primary**	1	93°C 1 min, 95°C 1 min
	5	94°C 1 min, 60°C 1 min, 72°C 1.5 min
	1	94°C 1 min, 25°C 3 min, ramping to 72°C in 3 min
	15	94°C 30 s, 63°C 1 min, 72°C 2.5 min
		94°C 30 s, 63°C 1 min, 72°C 2.5 min
		94°C 30 s, 44°C 1 min, 72°C 2.5 min
	1	72°C 8 min, 4°C forever
**Secondary**	12	94°C 30 s, 63°C 1 min, 72°C 2.5 min
		94°C 30 s, 63°C 1 min, 72°C 2.5 min
		94°C 30 s, 44°C 1 min, 72°C 2.5 min
	1	72°C 8 min, 4°C forever
**Tertiary**	20	94°C 30 s, 63°C 1 min, 72°C 2.5 min
		94°C 30 s, 63°C 1 min, 72°C 2.5 min
		94°C 30 s, 44°C 1 min, 72°C 2.5 min
	1	72°C 8 min, 4°C forever
**Primers**	Size (bp)	Sequences^a^
**AD1**	15	5' NTCGASTWTSGWGTT3'
**AD2**	16	5' NGTCGASWGANAWGAA3'
**AD3**	16	5' WGTGNAGWANCANAGA3'
**dSP1**	24	5' ATTGGGAACGACGCTGCCGTTATA 3'
**dSP2**	24	5' ACGGAATACCCATGACGATTTTGG3'
**dSP3**	24	5' CTGGCGTTGTGATTCGTGGTTTGT 3'
**uSP1**	24	5' CGGCTTCCAAAATCGTCATGGGTA 3'
**uSP2**	24	5' TATAACGGCAGCGTCGTTCCCAAT 3'
**uSP3**	24	5' CAGCCGCGTACGTGAACAAAAATG 3'
**uSP4**	24	5' CAGCTCCGGCCAATCTGAGGGTAA 3'
**uSP5**	24	5' ATCAAGGGGATGCGCTCGCAGACA 3'
**uSP6**	24	5' CGGAAACCTTTCACCTGCCAGCAA 3'

^a^N = A/G/C/T, S = C/G, W = A/T.

**Figure 1:**
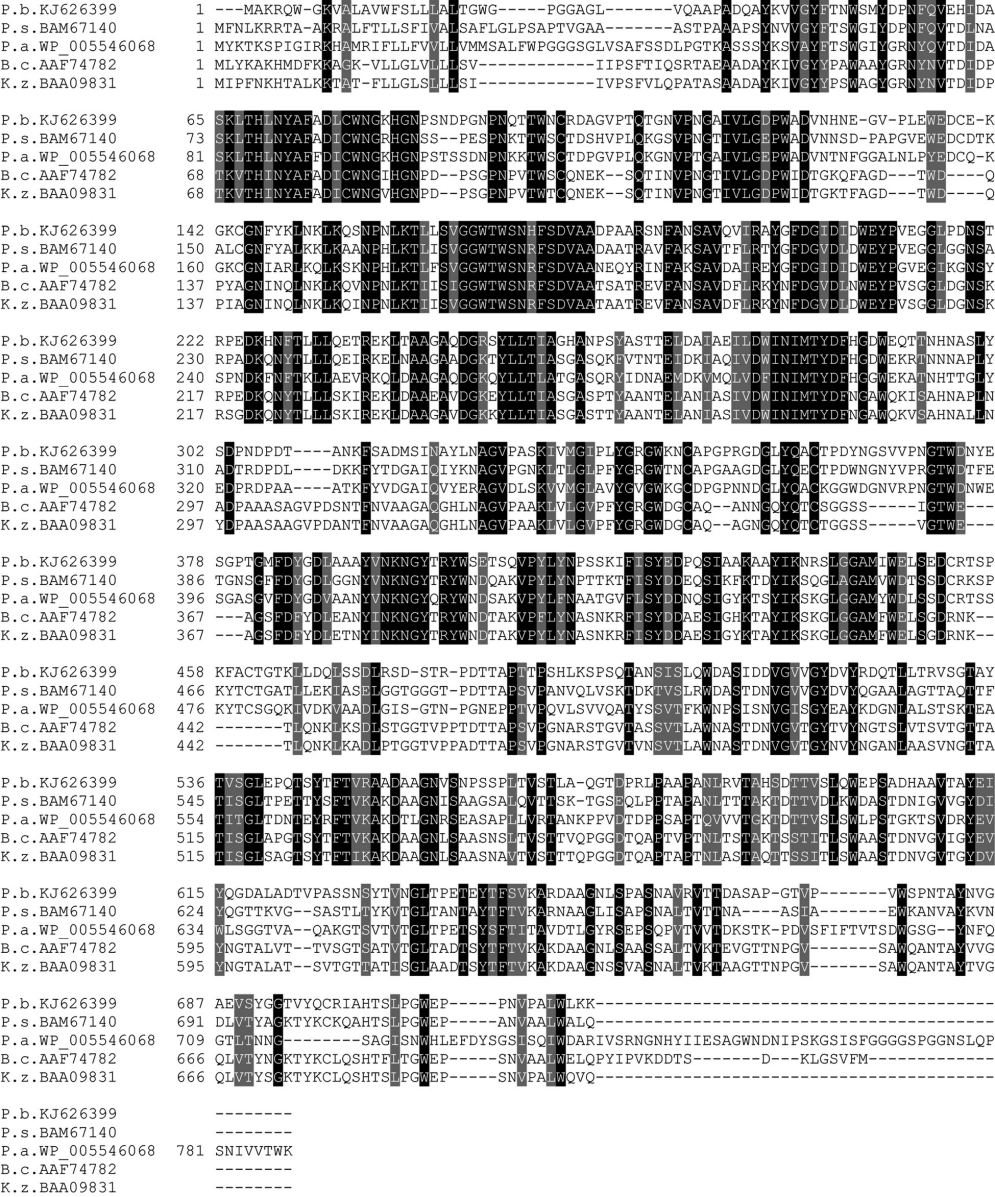
**Sequence alignment of PbChi74 from** ***P. barengoltzii*** **with other bacterial chitinases.** Numbers on the left are the positions of the first amino acids in each line. The other listed sequences include the chitinases from *Paenibacillus* sp. FPU7 (BAM67140), *Paenibacillus alvei* (WP_005546068), *Bacillus circulans* (AAF74782), and *Kurthia zopfii* (BAA09831).

Multiple amino acid sequence alignments revealed that PbChi74 showed the highest identity of 57% with a chitinase from *Paenibacillus* sp. FPU7 (BAM67140 [11]) followed by the chitinases from *Paenibacillus alvei* (WP_005546068, 54% identity), *Bacillus circulans* (AAF74782, 53% identity), and *Kurthia zopfii* (BAA09831, 53% identity) (Figure 1). Domain structure prediction analysis indicated that PbChi74 was a multimodular protein having a GH family 18 catalytic domain (TIM barrel), two fibronectin type III domains, and a carbohydrate binding domain (CBM-5/12) involved in substrate binding. Thus, PbChi74 may be a novel member of GH family 18 chitinases.

### Enzyme expression and purification

**Figure 2:**
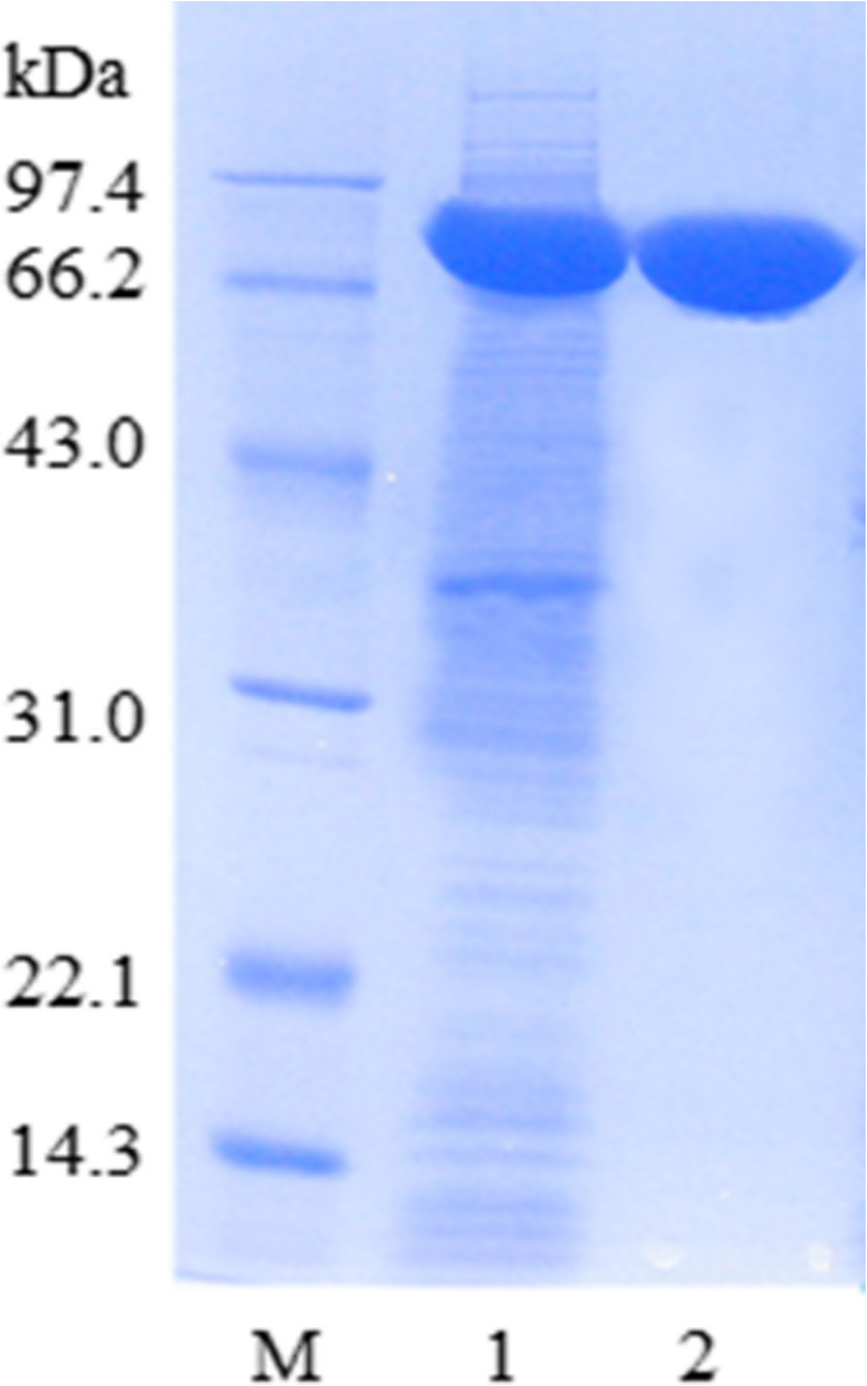
**SDS-PAGE analysis of proteins during the purification process of PbChi74 from** ***P. barengoltzii*** **expressed in** ***E. coli*** *.* Lane M, low molecular weight protein standards; lane 1, crude enzyme (supernatant); lane 2, purified chitinase after Ni-NTA column.

The amplified PbChi74-coding DNA fragment was inserted into the pET28a (+) plasmid with both *Bam*HI and *Xho*I restriction sites, resulting in an expression vector designated *PbChi74*-pET28, and then expressed in *E. coli* as an active protein. The recombinant chitinase (PbChi74) was purified to apparent homogeneity with a purification fold of 2.2 and a recovery yield of 57.9% (Table 2). The denatured molecular mass of the enzyme was estimated to be 74.6 kDa on SDS-PAGE (Figure 2), while the native molecular mass was determined to be 74.3 kDa by gel filtration (data not shown), suggesting that the enzyme is a monomer.

**Table 2:** **Purification summary of PbChi74 from** ***P. barengoltzii***

**Purification step**	**Total activity (U)** ^**a**^	**Total protein (mg)** ^**b**^	**Specific activity (U mg** ^**-1**^ **)**	**Purification factor (fold)**	**Recovery yield (%)**
**Crude enzyme**	563.7	63.4	8.8	1	100
**Ni-NTA agarose**	326.6	16.4	19.9	2.2	57.9

^a^Enzyme activity was measured at 65°C in 50 mM citrate buffer (pH 4.5) using 1% (w/v) of colloidal chitin as the substrate.

^b^Protein concentration was measured by the Lowry method [41] using BSA as the standard.

### Characterization of PbChi74

**Figure 3:**
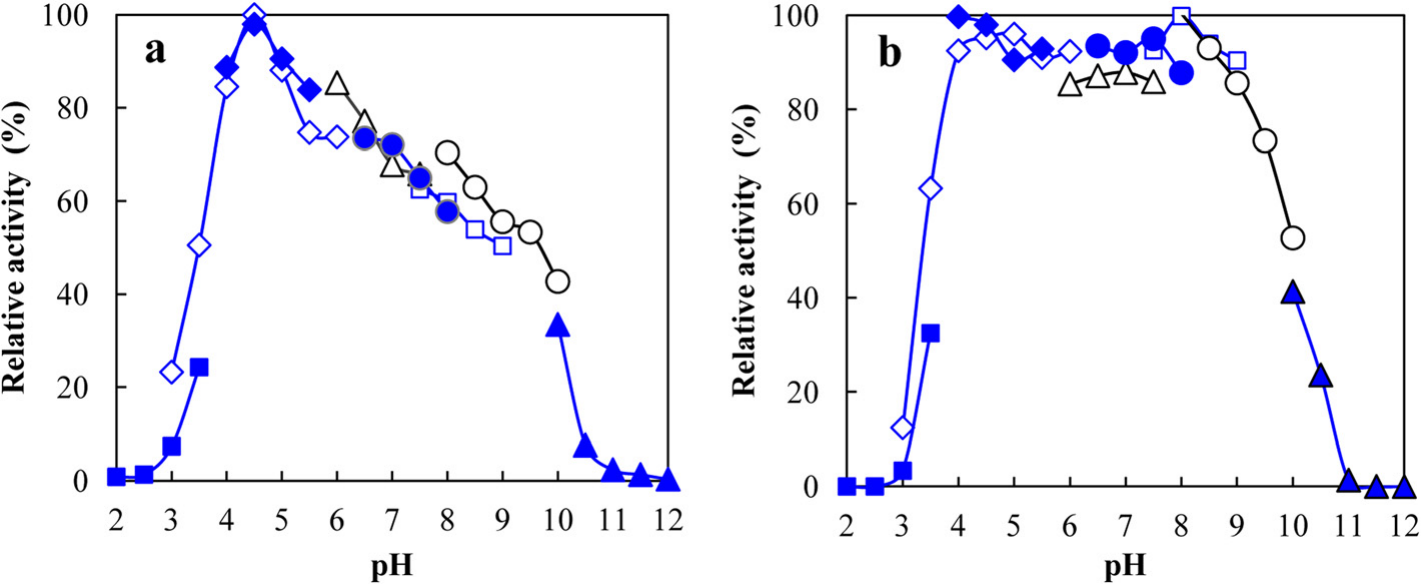
**Optimal pH (a) and pH stability (b) of the purified PbChi74 from** ***P. barengoltzii*** **.** The optimal pH was determined in 50 mM of various buffers within pH 2.0-12.0. The buffers used were: glycine-HCl (solid squares) (pH 2.0-3.5), sodium citrate (open diamonds) (pH 3.0-6.0), acetate (solid diamonds) (pH 4.0-5.5), sodium phosphate (open triangles) (pH 6.0-7.5), Tris-HCl (solid circles) (pH 6.5-8.0), CHES (open squares) (pH 7.5-9.0), MOPS (open circles) (pH 8.0-10.0), and glycine-NaOH (solid triangles) (pH 10.0-12.0). To determine pH stability, the enzyme aliquots were incubated in different buffers mentioned above at 30°C for 30 min and then the residual activities were measured.

**Figure 4:**
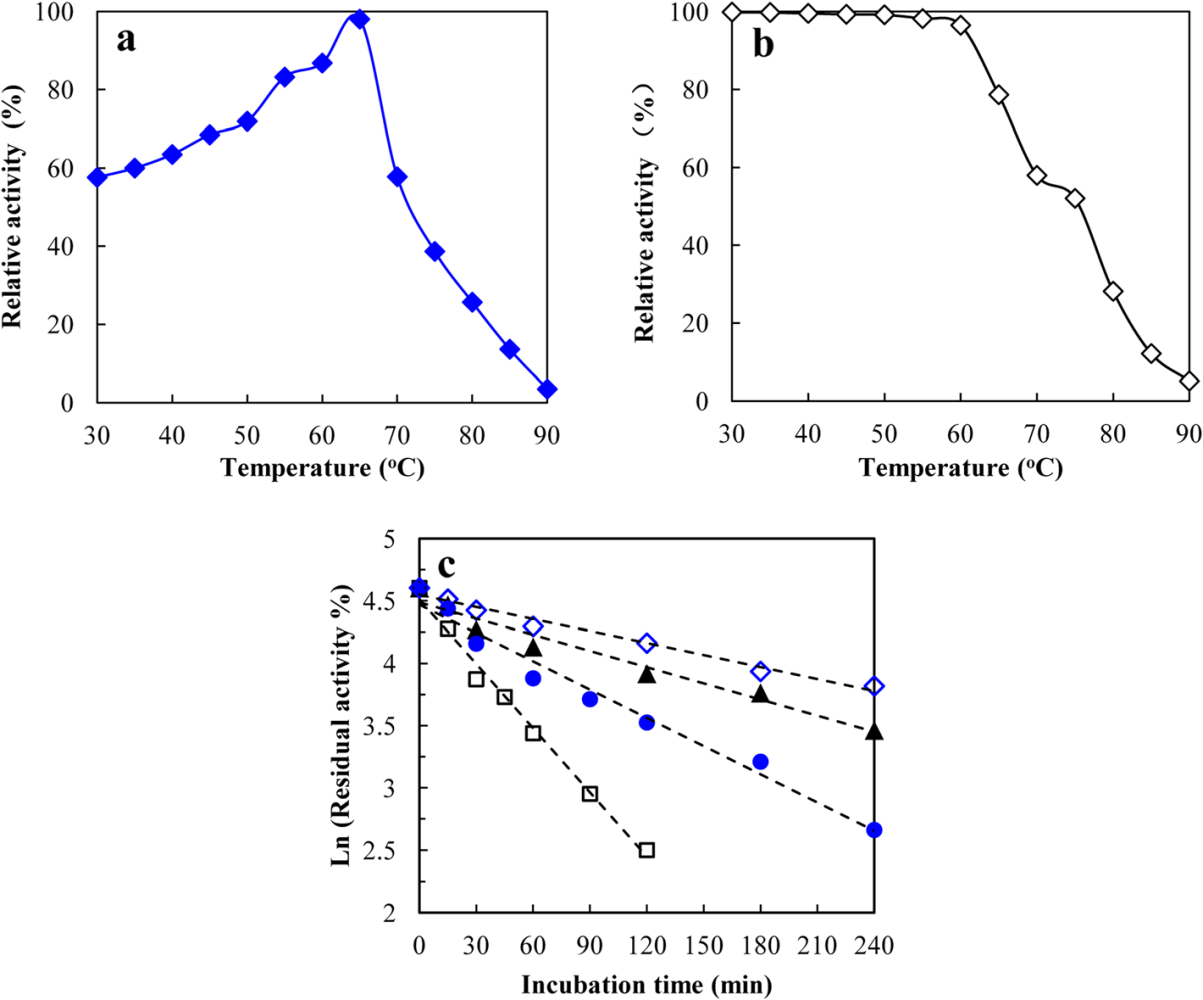
**Optimal temperature (a), thermal stability (b), and thermal inactivation (c) of the purified PbChi74 from** ***P. barengoltzii.*** The temperature optimum was determined at different temperatures (30-80°C) in 50 mM sodium citrate (pH 4.5). For determination of thermostability, the residual activity was measured in 50 mM sodium citrate (pH 4.5) at 65°C after the enzyme was treated for 30 min at different temperatures. For determination of thermal inactivation, the enzyme was incubated at 65°C (open diamonds), 70°C (solid triangles), 75°C (solid circles), and 80°C (open squares) for 4 h.

PbChi74 was most active at pH 4.5 (Figure 3a), and exhibited good stability within pH 4.0-9.0, since more than 80% of the activity was retained after the enzyme was treated in different buffers (pH 4.0-9.0) for 30 min (Figure 3b). The optimal temperature of PbChi74 was found to be 65°C (Figure 4a), and it was stable up to 60°C, as the residual activity was up to 96.5% of its original activity after treatment at this temperature for 30 min (Figure 4b). The half-lives of the enzyme at 65, 70, 75, and 80°C were 211.7, 144.0, 79.7, and 35.2 min, respectively (Figure 4c).

### Substrate specificity and kinetic parameters of PbChi74

PbChi74 exhibited the highest activity toward glycol chitin (22.2 U mg^-1^) followed by colloidal chitin (19.9 U mg^-1^) among the tested polysaccharides (Table 3). It also showed slight activities toward other polysaccharides such as powdery chitin (0.6 U mg^-1^), carboxymethylcellulose (CMC) (0.5 U mg^-1^), and chitosan (0.3 U mg^-1^) (Table 3). In addition, PbChi74 showed high activities on the tested *N*-acetyl COSs including (GlcNAc)_5_ (198.4 U mg^-1^), (GlcNAc)_4_ (68.0 U mg^-1^), (GlcNAc)_3_ (23.5 U mg^-1^), and (GlcNAc)_2_ (2.6 U mg^-1^). Unexpectedly, the enzyme showed relative high activity toward *p*NP-NAG (6.4 U mg^-1^), indicating that PbChi74 has *N*-acetyl-glucosaminidase activity (Table 3).

**Table 3:** **Substrate specificity of the purified PbChi74 from** ***P. barengoltzii***

**Substrate**	**Specific activity (U mg** ^**-1**^ **)** ^**a**^
**Colloidal chitin**	19.9 ± 0.2
**Glycol chitin**	22.2 ± 0.2
**Powdery chitin**	0.6 ± 0.1
**CMC**	0.5 ± 0.1
**Chitosan** ^**b**^	0.3 ± 0.05
***p*** **NP-NAG**	6.4 ± 0.1
**(GlcNAc)**_2_	2.6 ± 0.3
**(GlcNAc)**_3_	23.5 ± 0.8
**(GlcNAc)**_4_	68.0 ± 2.1
**(GlcNAc)**_5_	198.4 ± 7.9

^a^The enzyme activity was determined at 65°C in 50 mM sodium citrate buffer (pH 4.5).

^b^The degree of deacetylation of chitosan used is 85%.

The kinetic parameters (*K*_m_ and *V*_max_) of PbChi74 toward colloidal chitin and glycol chitin were determined to be 2.4 mg mL^-1^ and 23.22 μmol min^-1^ mg^-1^, 1.84 mg mL^-1^ and 23.4 μmol min^-1^ mg^-1^, respectively (Table 4).

**Table 4:** **Kinetic parameters of PbChi74 from** ***P. barengoltzii*** ^***a***^

**Substrate**	***V***_max_ **(μmol min** ^**-1**^ **mg** ^**-1**^ **)**	***K***_m_ **(mg mL** ^**-1**^ **)**	***k***_cat_ **(s** ^**-1**^ **)**	***k***_cat_ **/** ***K***_m_ **(mg** ^**-1**^ **s** ^**-1**^ **)**
**Colloidal chitin**	23.22	2.4	0.028	0.011
**Glycol chitin**	23.4	1.84	0.029	0.016

^a^The kinetic parameters were determined at 65°C in 50 mM sodium citrate buffer (pH 4.5) for 5 min.

### Hydrolysis pattern of the PbChi74

**Figure 5:**
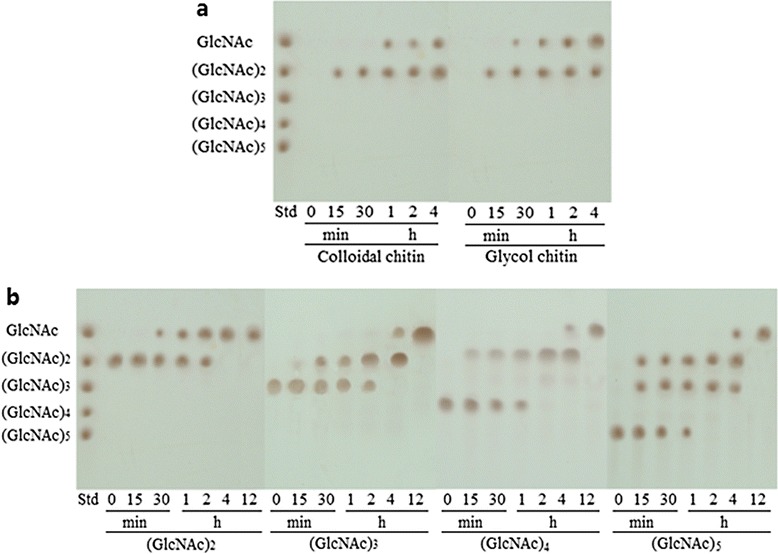
**Hydrolysis pattern of PbChi74 toward colloidal chitin (a) and** ***N*** **-acetyl chitooligosaccharides with DP 2-5 (b).** The reactions were performed at 50°C in 50 mM citrate buffer (pH 4.5), and aliquots were withdrawn at different time intervals and analyzed by thin layer chromatography (TLC).

PbChi74 hydrolyzed colloidal chitin or glycol chitin to yield only (GlcNAc)_2_ at the initial hydrolysis stage (0 to 15 min), and GlcNAc was then accumulated with the extension of the incubation time (15 to 240 min). In order to clarify whether (GlcNAc)_2_ was released directly from the non-reducing end of colloidal chitin, the hydrolysis products of colloidal chitin at the early hydrolysis stage (2, 4, 8, and 12 min) were further analyzed by HPLC. The results showed that PbChi74 hydrolyzed colloidal chitin to yield mainly (GlcNAc)_2_ and trace amounts of GlcNAc (data not shown). The results suggest that PbChi74 should be an exochitinase. At the end of the reaction, (GlcNAc)_2_ and GlcNAc were the major hydrolysis products (Figure 5a). In order to further detect the hydrolysis pattern of PbChi74, *N*-acetyl chitooligosaccharides (DP 2-5) were hydrolyzed by PbChi74 (Figure 5b). All of the GlcNAc oligomers were completely converted to the monomer GlcNAc at the end of the reactions, confirming that PbChi74 possesses β-*N*-acetylglucosaminidase activity. Therefore, PbChi74 is an exochitinase with β-*N*-acetylglucosaminidase activity.

### Conversion of colloidal chitin to GlcNAc by PbChi74 coupled with RmNAG

**Figure 6:**
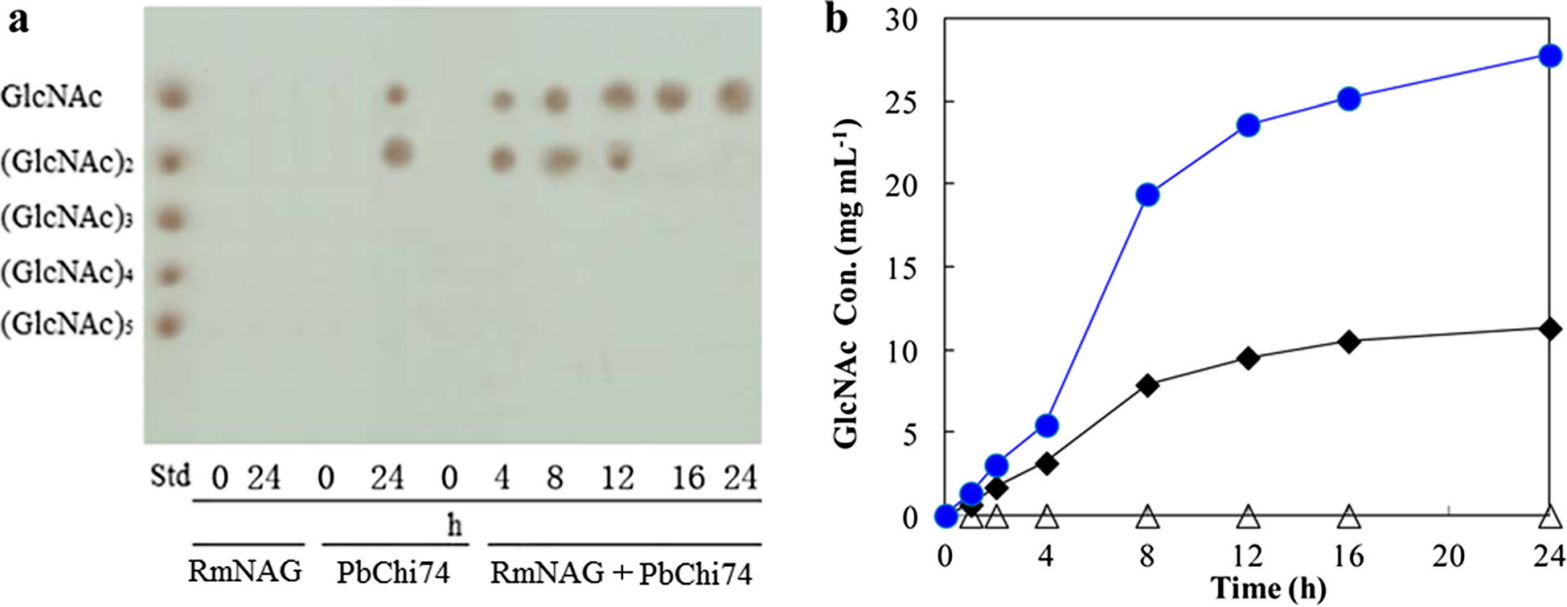
**TLC (a) and HPLC analysis (b) of hydrolysis products from colloidal chitin by the purified PbChi74 coupled with RmNAG.** 5 U mL^-1^ of PbChi74 (solid diamonds) or 1 U mL^-1^ of RmNAG (open triangles) or 5 U mL^-1^ of PbChi74 together with 1 U mL^-1^ of RmNAG (solid circles) were added to 30 mg mL^-1^ of colloidal chitin solution in 50 mM citrate buffer (pH 4.5), and incubated at 45°C for 24 h, separately. Aliquots taken at different times were analyzed by TLC and HPLC, respectively.

TLC analysis revealed that PbChi74 alone hydrolyzed colloidal chitin to yield mainly (GlcNAc)_2_ and GlcNAc, while RmNAG alone could not hydrolyze colloidal chitin (Figure 6a). However, PbChi74 coupled with RmNAG efficiently converted colloidal chitin to GlcNAc as the sole end product after 24 h incubation (Figure 6a). The highest chitin conversion ratio of 92.6% at a GlcNAc concentration of 27.8 mg mL^-1^ was obtained (Figure 6b).

## Discussion

Chitinases have drawn much attention in recent years due to their great potential in industrial applications [1,5]. To date, a number of microbial endochitinases have been identified and characterized [1,4], whereas there is still only limited information on exochitinases [7,16,20,28]. Furthermore, no exochitinases have been reported from *Paenibacillus* sp. Here, for the first time, we describe gene cloning, expression, characterization and application of a novel exochitinase from *Paenibacillus barengoltzii*.

A chitinase gene (*PbChi74*) from *P. barengoltzii* was cloned and functionally expressed in *E. coli*. PbChi74 had a GH family 18 catalytic domain and shared the highest identity of 57% with a chitinase from *Paenibacillus* sp. FPU7 [11]. The molecular masses of most reported chitinases from *Paenibacillus* species are in the range of 38 to 153 kDa [11]. The purified PbChi74 in the present study had a molecular mass of 74.6 kDa (Figure 2), which is different from those of chitinases from other *Paenibacillus* species, including *Paenibacillus* sp. FPU-7 (61, 78, 82 87, 97, 122, and 153 kDa [11]), *P. azotofixans* YUPP-5 (70 kDa [21]), and *Paenibacillus* sp. D1 (56.6 kDa [3]). Additionally, the molecular mass of PbChi74 is different from those of other exochitinases from microorganisms such as *Microbispora* sp. V2 (35 kDa [20]), *Streptomyces violaceusniger* (56.5 kDa [28]), *Bacillus* sp. DAU101 (66 kDa [16]), and *Thermococcus chitonophagus* (90 kDa [7]). Thus, PbChi74 should be a novel member of GH family 18 chitinases.

PbChi74 was an acidic chitinase with an optimal pH of 4.5 (Figure 3a). This value is lower than that of a chitinase from *Paenibacillus* sp. D1 (pH 5.0 [3]) and those of several exochitinases including the enzymes from *S. violaceusniger* (pH 5.0 [28]) and *Bacillus* sp. DAU101 (pH 7.5 [16]), but higher than that of an exochitinase from *Microbispora* sp. V2 (pH 3.0 [20]). It showed good pH stability within pH 4.0-9.0 (Figure 3b), which is a wider range than those of most other chitinases from bacteria such as *Halobacterium salinarum* (pH 6.0-8.5 [29]) and *S. violaceusniger* (pH 4.0-8.0 [28]). PbChi74 was most active at 65°C (Figure 4a), which is only close to the value for an exochitinase from *T. chitonophagus* (80°C [7]) among exochitinases, but is much higher than those of most other chitinases which have optimal temperatures in the range of 30-50°C [3,4,28]. However, this value is lower than those of several chitinases from *Bacillus* sp*.* (75°C [30]) and *Thermococcus kodakaraensis* KOD1 (ChiA5, 85°C and ChiA4, 90°C [31]). In addition, PbChi74 showed good thermostability at high temperatures, with half-lives of 212, 144, and 80 min at 70, 75, and 80°C, respectively (Figure 4c). The high temperature optimum and good thermal stability are advantageous for potential applications in recycling of biodegradable chitin wastes.

The specific activities of PbChi74 toward colloidal chitin (19.9 U mg^-1^) and glycol chitin (22.2 U mg^-1^) are much higher than those of most other reported chitinases [8,11,16,17,20,28]. Interestingly, PbChi74 exhibited high specific activity toward *p*NP-NAG (6.4 U mg^-1^), while most other chitinases have no or trace β-*N*-acetylglucosaminidase activities [32]. The action patterns of PbChi74 were investigated by analyzing the hydrolysis products of colloidal chitin and *N*-acetyl COSs (DP 2-5). PbChi74 efficiently released (GlcNAc)_2_ from the colloidal chitin, G3, G4, and G5 without formation of GlcNAc at the initial stage, suggesting that it is an exo-type chitinase. At the end of the hydrolysis process, (GlcNAc)_2_ was further converted to GlcNAc, confirming that PbChi74 has β-*N*-acetylglucosaminidase activity. The hydrolysis property of PbChi74 is similar to that of a bifunctional chitinase (Chisb) from *Bacillus* sp. DAU101 [16] and an exochitinase from *T. kodakaraensis* KOD1 [31]. Most other GH family 18 exochitinases exhibit a processive mode of action to release (GlcNAc)_2_ as the major final product, while they cannot hydrolyze (GlcNAc)_2_ to GlcNAc [33,34]. The unique property of PbChi74 may make the enzyme a good candidate for the conversion of chitin to GlcNAc.

GlcNAc, the structural monomer of chitin, has long been used as a valuable pharmacological agent in the treatment of a wide variety of ailments and as a functional additive in food production [2,35]. Traditionally, GlcNAc has been produced by acid hydrolysis of chitin, which results in environmental pollution. Hence, a “green” bioprocess for GlcNAc production is of great value. Usually, the complete conversion of chitin for GlcNAc production requires the synergistic action of chitinase and β-*N*-acetylglucosaminidase [22]. As PbChi74 has both chitinase and GlcNAcase activities, it was further investigated for GlcNAc production from chitin in this study. PbChi74 could not completely convert colloidal chitin to GlcNAc (Figure 6a), possibly because the β-*N*-acetylglucosaminidase activity is not sufficient. Co-action of PbChi74 and RmNAG for the hydrolysis of colloidal chitin significantly improved the GlcNAc production and resulted in GlcNAc as the only end product (about 100%, Figure 6), yielding 27.8 mg mL^-1^ with a high conversion ratio of up to 92.6% after a 24-h incubation. The concentration of generated GlcNAc is much higher than those of previous reports (3.46 mg mL^-1^ [36]; 4.4 mg mL^-1^ [35]; 7.5 mg mL^-1^ [37]; 9.5 mg mL^-1^ [24]; 15.4 mg mL^-1^ [35]; 21.3 mL^-1^ [25]). The conversion ratio is also higher than those of most other reports (73% [23]; 64 to 77% [35]; 85% [37]), and is only near to that of the report from Kuk *et al.* (94.9% [24]). It is noteworthy that the reaction time (24 h) in the present study is much shorter than those of previous reports (2 d [25], 2012; 4 d [36]; 6 d [37]; 10 d [35]). Therefore, the exochitinase PbChi74 could be a good candidate for GlcNAc production. The economic utilization of chitin as a feedstock for the bioethanol production would represent a profound shift in industrial carbon utilization, allowing sustainable resources to substitute for petroleum-based products, in which the biopolymer must first be broken down into constituent mono sugars that can be more easily converted in biological processes before its exploitation as a source material [38]. Hence, the hydrolytic property of PbChi74 may be beneficial for bioethanol production from chitin materials.

## Conclusions

A novel exochitinase (PbChi74) from *Paenibacillus barengoltzii* was gene cloned, expressed, and biochemically characterized. PbChi74 was a monomer with a molecular mass of 74.6 kDa. It was most active at pH 4.5 and 65°C. The enzyme exhibited high specific activity towards chitins, *N*-acetyl COSs (DP 3-5), and *p*NP-GlcNAc. It exhibited an exo-type cleavage pattern and a unique activity converting (GlcNAc)_2_ into its monomer. The enzyme together with RmNAG was efficiently used for the GlcNAc production from colloidal chitin. The excellent properties of the enzyme may give it great potential in chitin conversion.

## Methods

### Strains, plasmids, and reagents

The newly isolated marine bacterium *Paenibacillus barengoltzii* CAU904 was used in this study. The strain has been deposited in the China General Microbiological Culture Center (CGMCC) under accession number [CGMCC:9530]. *Escherichia coli* DH5α (Biomed, Beijing, China) and *E. coli* BL21 (Biomed) were used as host strains for DNA manipulation and gene expression, respectively. pET28a(+) was used as the expression vector (Novagen, Madison, WI, USA). Taq DNA polymerase and restriction endonuclease were obtained from TaKaRa (Shiga, Japan). T4 DNA ligase was purchased from New England Biolabs (Ipswich, MA, USA).

Chitin (from crab shells), glycol chitin, powdery chitin, carboxymethylcellulose (CMC), and *p*-nitrophenyl *N*-acetyl-β-D-glucosaminide (*p*NP-NAG) were obtained from Sigma Chemicals Co. (St. Louis, MO, USA). Chitosan with a deacetylation degree of 85% was obtained from Fluka (Buchs, Switzerland). *N*-acetyl chitooligosaccharides with degree of polymerization (DP) 2-5 were purchased from Seikagaku (Tokyo, Japan). Chelating Sepharose Ni-iminodiacetic acid (Ni-IDA) and Sephacryl S-200 HR resins were purchased from GE Healthcare (Piscataway, NJ, USA). Silica gel plates were obtained from E. Merck Co. (Darmstadt, Germany). All other chemicals used were of analytical grade unless otherwise stated.

### Cloning and sequence analysis of a chitinase gene

For isolation of genomic DNA, *P. barengoltzii* CAU904 was cultivated at 50°C for 3 days in a medium consisting of (g L^-1^): colloidal chitin 10, tryptone 5, yeast extract 5, K_2_HPO_4_ 0.87, KH_2_PO_4_ 0.68, MgSO_4_ 0.2, sea salt 5. The genomic DNA was isolated and used as the template for polymerase chain reaction (PCR) amplification. The partial core region of the chitinase gene was amplified using degenerate primers ChiF (5'ATHAAYATHATGACNTAYGA3') and ChiR (5'CCAGTACCGNKTRTANCCRTT3'), corresponding to the conserved motifs (INIMTYD and NGYK/TRYW, respectively) of GH family 18 chitinases. The PCR conditions were as follows: 5 min at 94°C, followed by 20 cycles of 95°C for 30 s, 55°C for 30 s, and 72°C for 1 min, with 1°C decrease in annealing temperature per cycle, then 15 cycles of 95°C for 30 s, 50°C for 30 s, and 72°C for 1 min, followed by a final extension of 10 min. The PCR products were purified and ligated into the pMD-18T vector for sequencing. A modified hiTAIL-PCR was used to obtain the full-length gene of chitinase from *P. barengoltzii* CAU904 [39]. hiTAIL-PCR was performed under the conditions listed in Table 1. The nested known sequence specific primers (SP) were designed accordingly and used to amplify the 5′ and 3′ flanking regions of the core region in consecutive reactions together with arbitrary degenerate (AD) primers. The full-length chitinase gene was obtained by conducting the hiTAIL-PCR twice with six SPs and three ADs (Table 1). Finally, the coding region of the gene without the signal peptide was amplified by PCR amplification with the primers Chi1BamHF (5'AAC**GGATCC**GCCGATCAGGCCTACAAG3', *Bam*HI restriction site is underlined) and Chi1XhoR (5'ACC**CTCGAG**TTTCAGCCATAACGCAGG3', *Xho*I restriction site is underlined) and an annealing temperature of 55°C. The gene has been submitted to NCBI GenBank under accession number [GenBank:KJ626399].

Nucleotide and deduced amino acid sequences were analyzed with the ExPASy Proteomics tools (http://www.expasy.org/tools). Database homology searches of nucleotide sequences were carried out using BLAST at the NCBI (http://blast.ncbi.nlm.nih.gov/Blast.cgi). The amino acid sequences were aligned by the ClustalW program (ftp://ftp-igbmc.u-strasbg.fr/pub/ClustalW/). The signal peptide was analyzed at the SignalP 3.0 server (http://www.cbs.dtu.dk/services/SignalP-3.0). A search analysis of conserved domain and signature sequences was carried out using Scan Prosite (http://prosite.expasy.org/scanprosite).

### Expression of the chitinase gene in *E. coli*

The PCR product amplified using the specific primers Chi1BamHF and Chi1XhoR was purified and subcloned into the pET28a(+) vector. The recombinant vectors were then transformed into *E. coli* BL21 (DE3) for protein expression. The positive clones were directly screened by colony PCR and cultured in LB media containing 50 μg mL^-1^ of kanamycin at 37°C in a shaker. After the optical density (OD_600_) of the culture medium was up to 0.6, isopropyl-β-D-thio-galactopyranoside (IPTG) was added at a final concentration of 1 mM for protein induction, and the culture was further grown at 30°C for 10 h.

### Purification of the recombinant chitinase (PbChi74)

The culture broth (300 mL) was harvested by centrifuging at 5,000 g for 10 min, and the precipitated cells were collected, resuspended in 50 mM Tris–HCl buffer (pH 8.0) and disrupted by ultrasonication. The cell debris was removed by centrifuging at 8,900 *g* for 10 min, and the supernatant was collected and used as the crude enzyme. The crude enzyme was loaded on a Ni-IDA column (1 × 10 cm) which was pre-equilibrated with buffer A (50 mM Tris–HCl buffer pH 8.0 containing 20 mM imidazole and 500 mM NaCl). The column was washed with 5 column volumes (CV) of buffer A followed by 5 CV of buffer B (50 mM Tris–HCl pH 8.0 containing 50 mM imidazole and 500 mM NaCl). After then, the bound proteins were eluted with buffer C (50 mM Tris–HCl buffer pH 8.0 containing 200 mM imidazole and 500 mM NaCl). The flow rate during the purification process was fixed at 1 mL min^-1^ unless otherwise specified. The eluted fractions showing chitinase activity were checked for purity by SDS-PAGE. The purified fractions were combined, concentrated, and buffer exchanged for 50 mM phosphate buffer (pH 6.0).

The native molecular mass of PbChi74 was estimated by gel filtration on a Sephacryl S-200 HR column (1 × 50 cm). The proteins were eluted with 50 mM phosphate buffer (pH 6.0) containing 150 mM NaCl at a flow rate of 0.3 mL min^-1^. The molecular mass standards used for calibration were phosphorylase b (97.2 kDa), fetuin from fetal calf serum (68.0 kDa), albumin from chicken egg white (45.0 kDa), and chymotrypsinogen A (25.7 kDa).

### Enzyme assay and SDS-PAGE analysis

The chitinase activity was assayed as described by Lee *et al.* [16] with minor modifications. The reaction mixture containing 0.1 mL of suitably diluted enzyme solution and 0.1 mL of 1% (w/v) colloidal chitin solution was incubated at 50°C for 30 min. The amount of released reducing sugars was then determined by the dinitrosalicylic acid (DNS) method [40]. One unit (U) of chitinase activity was defined as the amount of enzyme required to liberate 1 μmol of reducing sugars per minute under the above conditions using *N*-acetyl glucosamine (GlcNAc) as the standard. The protein concentration was estimated according to the method of Lowry *et al.* [41] using BSA (bovine serum albumin) as the standard. Specific activity was expressed as units per milligram of protein.

SDS-PAGE was performed according to the method of Laemmli [42] using 12.5% separation gel. Protein bands were visualized by staining with Coomassie Brilliant Blue R-250. The molecular mass standards (TaKaRa, Dalian, China) included phosphorylase b (97.2 kDa), albumin (66.4 kDa), ovalbumin (44.3 kDa), carbonic anhydrase (29.0 kDa), trypsin inhibitor (20.1 kDa), and lysozyme (14.3 kDa).

### Effect of pH and temperature on the activity and stability of the purified PbChi74

The effect of pH on the activity of PbChi74 was determined in various buffers (50 mM) within pH 2.0-12.0. The buffers used were glycine-HCl (pH 2.0-3.5), citrate (pH 3.0-6.0), acetate (pH 4.0-5.5), phosphate (pH 6.0-7.5), Tris-HCl (pH 7.5-9.0), MOPS (pH 6.5-8.0), CHES (pH 8.0-10.0), and glycine-NaOH (pH 10.0-12.0). To determine the pH stability, the enzyme samples were incubated in the above-mentioned buffers at 30°C for 30 min, and the residual activities were then measured by the standard enzyme assay.

The optimal temperature of PbChi74 was determined in 50 mM citrate buffer (pH 4.5) at different temperatures (30 to 80°C). For thermal stability estimation, the aliquots were pre-incubated in 50 mM citrate buffer (pH 4.5) at different temperatures (30 to 80°C) for 30 min, and the residual activities were then assayed. The thermal inactivation of the enzyme was estimated by incubating the enzyme in 50 mM acetate buffer pH 4.5 at different temperatures (65, 70, 75, and 80°C) for 4 h. Aliquots were taken at different time intervals, and their residual activities were measured according to the standard enzyme assay.

### Substrate specificity and kinetic parameters of the purified PbChi74

For substrate specificity, the enzyme activity of PbChi74 was determined in 50 mM citrate buffer (pH 4.5) at 65°C for 30 min using 1% (w/v) of different polysaccharides (colloidal chitin, carboxymethylcellulose, carboxymethyl chitin, glycol chitin), *N*-acetyl chitooligosaccharides with degree of polymerization (DP) 2-5 as well as *p*NP-NAG (2 mM). The products released from the *N*-acetyl chitooligosaccharides were analyzed by an HPLC Evaporative Light Scattering Detector (Agilent 1260 Series, Agilent Technologies, Santa Clara, CA, USA) equipped with a Cosmosil Sugar-D column (4.61 × 250 mm, Japan). The mobile phase used was acetonitrile/water (75:25, v/v), and the flow rate was set at 1 mL min^-1^. For *p*NP-GlcNAc used as the substrate, a reaction mixture containing 50 μL of enzyme solution, 100 μL of *p*NP-NAG (2 mM), and 50 μL of 200 mM citrate buffer (pH 4.5) was incubated at 65°C for 10 min. The reaction was terminated by the addition of 200 μL of 0.5 M NaOH solution, and then the amount of *p*NP released was determined by measuring the absorbance at 410 nm. One unit of enzyme activity was defined as the amount of enzyme required to liberate 1 μmol of reducing sugars or *p*NP per minute under the above assay conditions.

Kinetic parameters of the PbChi74 towards colloidal chitin and glycol chitin were determined by measuring the enzyme activity using different substrate concentrations in 50 mM citrate buffer (pH 4.5) at 65°C for 5 min. The *K*_m_ and *V*_max_ values were calculated using GraFit software.

### Hydrolysis pattern of the purified PbChi74

The hydrolysis properties of PbChi74 on colloidal chitin and *N*-acetyl chitooligosaccharides (DP 2-5) were analyzed by incubating 1% (w/v) of substrates in 50 mM citrate buffer (pH 4.5) with 1 U mL^-1^ of enzyme at 50°C for 4 h. Aliquots withdrawn at different time intervals were immediately boiled for 10 min to terminate the enzyme, and the hydrolysis products were then analyzed by thin layer chromatography (TLC) according to the method of Yang *et al.* [27]. To detect the hydrolysis pattern of PbChi74, the products from colloidal chitin at the initial stage were quantitatively analyzed by HPLC.

### Conversion of colloidal chitin to GlcNAc

The reaction mixture (600 mL), consisting of 3% (w/v) colloidal chitin dissolved in 50 mM phosphate buffer (pH 6.0), was incubated in the presence of 5.0 U mL^-1^ of PbChi74, or 1 U mL^-1^ of RmNAG or 5.0 U mL^-1^ of PbChi74 combined with 1 U mL^-1^ of RmNAG at 45°C for 24 h. The β-*N*-acetylglucosaminidase (RmNAG) from *Rhizomucor miehei* was prepared according to a previous study [27]. Samples were taken at different times and boiled for 5 min to terminate the enzymes. After centrifuging at 7000 *g* for 10 min, the supernatant was collected and qualitatively and quantitatively analyzed by TLC and HPLC, respectively. The conversion ratio is the percentage of released GlcNAc weight (mg) to initial total colloidal chitin weight (mg).

## Abbreviations

CHES: 2-(cyclohexylamino)ethanesulfonic acid
COSs: chitooligosaccharides
DP: degree of polymerization
GlcNAc: *N*-acetyl glucosamine
(GlcNAc)_2_: *N-*acetyl chitobiose
MOPS: 3-(*N*-morpholino) propanesulfonic acid
Ni-IDA: Ni-iminodiacetic acid
PbChi74: a 74-kDa chitinase from *Paenibacillus barengoltzii*
*p*NP-NAG: *p*-nitrophenyl *N*-acetyl-β-D-glucopyranoside
RmNAG: β-*N*-acetylglucosaminidase from *Rhizomucor miehei*
